# Trypanosomatid parasites in Austrian mosquitoes

**DOI:** 10.1371/journal.pone.0196052

**Published:** 2018-04-19

**Authors:** Ellen Schoener, Sarah Susanne Uebleis, Claudia Cuk, Michaela Nawratil, Adelheid G. Obwaller, Thomas Zechmeister, Karin Lebl, Jana Rádrová, Carina Zittra, Jan Votýpka, Hans-Peter Fuehrer

**Affiliations:** 1 Institute of Parasitology, Department of Pathobiology, University of Veterinary Medicine Vienna, Vienna, Austria; 2 Federal Ministry of Defence and Sports, Division of Science, Research and Development, Vienna, Austria; 3 Biological Station Lake Neusiedl, Burgenland, Austria; 4 Institute for Veterinary Public Health, Department for Farm Animals and Veterinary Public Health, University of Veterinary Medicine Vienna, Vienna, Austria; 5 Department of Parasitology, Faculty of Science, Charles University, Prague, Czechia; 6 Institute of Parasitology, Biology Centre of Czech Academy of Sciences, České Budĕjovice, Czechia; University of Ostrava, CZECH REPUBLIC

## Abstract

Trypanosomatid flagellates have not been studied in Austria in any detail. In this study, specific nested PCR, targeted on the ribosomal small subunit, was used to determine the occurrence and diversity of trypanosomatids in wild-caught mosquitoes sampled across Eastern Austria in the years 2014−2015. We collected a total of 29,975 mosquitoes of 19 species divided in 1680 pools. Of these, 298 (17.7%), representing 12 different mosquito species, were positive for trypanosomatid DNA. In total, seven trypanosomatid spp. were identified (three *Trypanosoma*, three *Crithidia* and one *Herpetomonas* species), with the highest parasite species diversity found in the mosquito host *Coquillettidia richiardii*. The most frequent parasite species belonged to the mammalian *Trypanosoma theileri*/*cervi* species complex (found in 105 pools; 6.3%). The avian species *T*. *culicavium* (found in 69 pools; 4.1%) was only detected in mosquitoes of the genus *Culex*, which corresponds to their preference for avian hosts. Monoxenous trypanosomatids of the genus *Crithidia* and *Herpetomonas* were found in 20 (1.3%) mosquito pools. One third (n = 98) of the trypanosomatid positive mosquito pools carried more than one parasite species. This is the first large scale study of trypanosomatid parasites in Austrian mosquitoes and our results are valuable in providing an overview of the diversity of these parasites in Austria.

## Introduction

Trypanosomatids are flagellates parasitizing both invertebrates and vertebrates [1–3]. They are evolutionary more ancestral than other protists [4] and there is evidence that the history of vertebrate trypanosomatid parasites, vectored by dipteran insects, reaches back to the Early Cretaceous [5]. Several genera of the family Trypanosomatidae are monoxenous (with only one host) parasites of dipteran insects, namely *Leptomonas*, *Crithida*, *Herpetomonas*, *Jaenimonas*, and *Strigomonas* [5, 6, 7] and the newly described *Sergeia* [8], *Angomonas* [9], *Kentomonas* [10] and *Zelonia* [11]. Monoxenous insect trypanosomatids remain neglected and represent a relatively obscure group within the family Trypanosomatidae. However, some members of the genus *Crithidia* were described in detail. In laboratory experiments they did not show any negative impact on their insect hosts, which are in most cases mosquitoes [12–14]. The members of the genus *Crithidia*, *Herpetomonas* and *Strigomonas* have been found in a wider variety of mosquitoes and other bloodsucking nematocerans [15–18] and advances in molecular genetics have aided in determining their systematic and taxonomy [2, 4, 19, 20]. These advances also lead to the proposal of several new genera, e.g., *Angomonas*, *Strigomonas*, *Kentomonas*, *Jaenimonas*, *Novymonas*, *Blechomonas* etc. [7, 9, 20–22]; and allowed the study of distribution, diversity and host specificity of monoxenous trypanosomatids which were published previously in [7, 23, 24].

There is a substantial support for the hypothesis that the dixenous life cycle emerged from the monoxenous one independently for representatives of the dixenous genera *Trypanosoma*, *Leishmania*, and *Phytomonas* [4, 7, 25, 26]. Therefore, monoxenous trypanosomatids of mosquitoes and other bloodsucking insects can represent a crucial evolutionary link which is important for the elucidation of the emergence of a dixenous parasite life cycle.

Today, trypanosomatids are known primarily as important dixenous parasites of vertebrates, transmitted by various invertebrate vectors. Several species of the genus *Trypanosoma* cause serious and even life-threatening diseases in livestock [27, 28] and two species, *T*. *brucei* s.l. [29, 30] and *T*. *cruzi* [31, 32], have a significant impact on human health. However, in the case of trypanosome infections, any serious impact on host health is rather an exception, and many trypanosome species occurring in wildlife and domestic animals may be considered as non-pathogenic parasites. Trypanosomes are common parasites of fish [33], birds, such as *T*. *avium* s.l. [16, 34–38], and of ungulates (especially domestic cattle), like the *T*. *theileri* complex [20, 39–41]. Both the *T*. *avium* and *T*. *theileri* species complexes are cosmopolitan and found worldwide [40, 42, 43].

Various dipteran insects have been identified as competent vectors of different bird trypanosome species by experimental infections, with *T*. *avium* transmitted by blackflies (*Similium* spp.) [35, 44], *T*. *corvi* by hippoboscid flies (*Ornithomyia* spp.) [37, 45], and the *T*. *bennetti* group by biting midges (Ceratopogonidae) [46]. In 2012, a new trypanosome species, *T*. *culicavium*, was described in Central Europe, and appears to be a parasite of insectivorous passerine birds with *Culex* mosquitoes as a vector [47].

Insect-borne trypanosomes found in Europe develop in the alimentary tract of bloodsucking insects [48] and are transmitted to vertebrates either by regurgitation of intestinal content [49], faecal matter deposited at the bite site [44] or by ingestion of the insect [47]. In the vertebrate hosts, these parasites can be found in blood [40, 50], bone marrow [51] or inner organs [52]. In general, these trypanosomes are not regarded as pathogenic for the vertebrate hosts and *T*. *theileri-*like parasites and avian trypanosomes do not appear to overtly affect their hosts [40, 50, 52]. In the insect vectors, however, parasites can have a much larger impact, e.g. due to the blockage and destruction of the stomodeal valve facilitating the parasite transmission to the vertebrate host [49]. Host specificity of trypanosomes in vertebrates depends on the species and some have very broad host spectra like *T*. *avium* s.l., which has been found to infect a wide variety of bird orders and families [35, 38, 51].

Austrian mosquitoes have not been examined for trypanosomatid parasites before. We therefore screened female mosquitoes collected over two years in three Eastern Austrian provinces, namely Burgenland, Lower Austria and Vienna, to gain an overview which mosquito-borne trypanosomatids are present in the area, as well as to determine parasite diversity and prevalence in different mosquito species.

## Material and methods

Trypanosomatid DNA for the study was obtained from mosquitoes sampled during a monitoring effort across the three provinces of Eastern Austria (Burgenland, Lower Austria, and Vienna) at 35 permanent and 25 non-permanent trapping sites. These sites were on public as well as private land, which was entered with the permission of the owners. Citizen Scientists in Lower Austria and Burgenland assisted with the sampling effort. At permanent sampling sites, mosquitoes were collected for a 24 hour time period on a regular basis every second week from April to October in 2014 and 2015, using BG- Sentinel traps (Biogents, Regensburg, Germany) equipped with bottled carbon dioxide (Air Liquide, Schwechat, Austria) as attractant. Non-permanent sampling sites were sampled at least once and up to six times over a 24 hour period during the summer months using CO_2_-baited BG-Sentinel traps as above or by hand aspirators. All mosquitoes were stored at −80°C until further procedure.

Morphological identification of mosquito species was performed using the identification key of Becker et al. [53] and females were pooled by species, collection site and date, with a maximum number of 50 individuals. In 2014, three legs of each individual of *Cx*. *pipiens* s.l. / *Cx*. *torrentium* were taken and processed individually to identify the species/biotypes genetically in the frame of another project [54]. These mosquitoes were pooled after genetic identification which allowed us to determine the trypanosomatid parasite incidence in different biotypes of this species complex in more detail.

**Fig 1:**
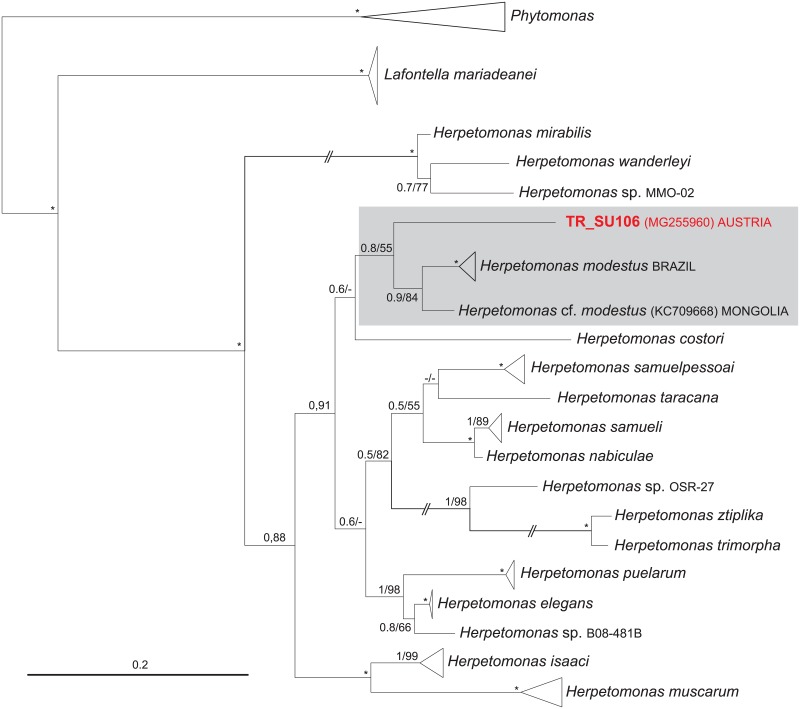
An SSU rDNA-based Bayesian phylogenetic tree representing the most likely new *Herpetomonas* species obtained from a mosquito collected in Eastern Austria. Bootstrap values from Bayesian posterior probabilities (5 million generations) and bootstrap percentages for maximum-likelihood (PhyML) analysis (1,000 replicates) are shown at the nodes; dashes indicate <50% bootstrap support or different topology; asterisks mark branches with maximal statistical support; double-crossed branches are 50% of the original length. The tree was rooted with five sequences of *Phytomonas* spp., the closest relative of the genus *Herpetomonas*. Parasite names, names of strains or GenBank accession numbers are given; the branch lengths are drawn proportionally to the amount of changes (scale bar).

For amplifying trypanosomatid parasite DNA, each DNA sample was then subjected to nested PCR, described by [55] without modification. The used primers target a ~2000 bp fragment of the ribosomal small subunit (SSU) gene. Obtained sequences were viewed and aligned using the software Geneious, version 10.0.6 [56]. Then the sequences were compared for similarity to sequences available on the GenBank^®^ database. In the case of SSU rRNA gene sequence (Acc. No.: MG255960) of the most likely new *Herpetomonas* species (TR_SU106), a phylogenetic tree was constructed using all available sequences of *Herpetomonas* species retrieved from GenBank with *Phytomonas* spp. as an outgroup (Fig 1). Alignments for phylogenetic analysis were generated in Kalign [57]; the ambiguously aligned positions in the trimmed alignment were removed manually in BioEdit (Ibis Therapeutics, Carlsbad, US). The final dataset contained 46 taxa and 1,988 nucleotide positions. Analyses were done in MrBayes [58] and PhyML [59] with model optimization in ModelTest [60], version 3.06. A general time-reversible substitution model with a mixed model for among-site rate variation (GTR + Γ + I) was chosen as the best fitting model of sequence evolution. Bootstrap analyses involved heuristic searches with 1,000 replicates (ML). Bayesian inference was accomplished in MrBayes 3.2.2 with analysis run for 5 million generations with covarion and sampling every 100 generations. Other parameters were left in their default states.

### Minimum infection rate

To evaluate the infection rate of the collected mosquitoes, the minimum infection rate (MIR) of each mosquito species was calculated. If a mosquito pool was positive for trypanosomatid DNA, it was assumed that the pool contained at least one positive individual. Therefore, MIR (percentage) was calculated as follows:

$$\text{MIR}\;(\%)={\text{n}}_{\;(\text{PCR}\;\text{positive}\;\text{pools})}/{\text{n}\;}_{\left(\text{total}\;\text{analysed}\;\text{mosquitoes}\right)}\times 100$$

## Results

A total of 29,975 mosquitoes, belonging to 19 species and five genera, were collected in Vienna and Eastern Austria in the years 2014 and 2015 (S1 Fig).

From these, 1680 pools were created using up to 50 mosquito females separated by species, time and site of sampling. A total of 298 (17.7%) mosquito pools were positive for trypanosomatid DNA (S1 Table). Of these, 243 pools (82.1%) belonged to 14 identified mosquito species and forma, whereas 53 pools (17.9%) were of unidentified individuals of the genera *Aedes*/*Ochlerotatus*, *Culex*, and *Anopheles*. Pools positive for trypanosomatids were of the following mosquito taxa: *Ae*. *cinereus/geminus*, *Ae*. *vexans*, *An*. *maculipennis* complex, *An*. *plumbeus*, *Cq*. *richiardii*, *Cx*. *pipiens* s.l. and *Cx*. *torrentium* (unspecified to forma level), *Cx*. *pipiens* f. *pipiens*, *Cx*. *pipiens* f. *molestus*, *Cx*. *pipiens* f. *pipiens*/*molestus* hybrid, *Cx*. *torrentium*, *Cx*. *martinii*, *Cx*. *modestus*, *Oc*. *geniculatus*, and *Oc*. *sticticus*.

Trypanosomid parasites were not found in the following 15 species: *Anopheles algeriensis*, *An*. *claviger*, *An*. *hyrcanus*, *Cs*. *annulata*, *Cx*. *territans*, *Oc*. *cantans*, *Oc*. *caspius*, *Oc*. *cataphylla*, *Oc*. *communis*, *Oc*. *flavescens*, *Oc*. *intrudens*, *Oc*. *japonicus*, *Oc*. *leucomelas*, *Oc*. *rusticus* and *Uranotaenia unguiculata*.

### Trypanosomatid parasite diversity

The most common trypanosomatid species found in the tested mosquito pools were trypanosomes belonging to the *Trypanosoma theileri*/*cervi* complex (No. of positive pools = 105, which represents 35.5% of all positive pools and 6.3% of all tested pools) and *T*. *culicavium* (n = 69; 23.2% / 4.1%), followed by *T*. *avium* s.l. (n = 3; 1.0% / 0.2%) and monoxenous species belonging to the genus *Crithidia* and *Herpetomonas* (n = 20; 7.0% / 1.3%). A total of three dixenous and four monoxenous trypanosomatid species were identified by the analysis of their SSU (S1 Table and Fig 1). One third of the examined mosquito pools positive for trypanosomatid DNA (n = 98; 33.1%) carried more than one parasite species, as could be seen on the electropherogram where different peaks superimposed on each other. The mosquito species with the highest diversity of different trypanosomatid parasites was *Cq*. *richiardii*, in which we found the *T*. *theileri* complex and all four detected species of monoxenous trypanosomatids (S1 Table). The trypanosome species *T*. *culicavium* was only detected in mosquitoes of the genus *Culex* and the species *T*. *avium* s.l. was found only in 2014. Since the whole bodies of mosquitoes were used in pools, and no dissection and microscopy was performed, it was not possible to assert competent vector status of trypanosomes on the sampled mosquito species.

The BLAST search of the GenBank database presented a vast majority of our received sequences with 100% sequence identity to previously published sequences of three trypanosomes, *T*. *culicavium* (MG255959), *T*. *avium* s.l. (one genotype, MG255950) and the *T*. *theileri*/*cervi* complex (five genotypes, MG255951, MG255952, MG255953, MG255954, MG255958), and three crithidia species, *Crithidia fasciculata* (MG255955), *C*. *brevicula* (MG255956), and *C*. *pragensis* (MG255957). The only exception is one single sequence, TR_SU106 (MG255960), found in *Cq*. *richiardii*, which represents a potential new species of the genus *Herpetomonas*. The position on the phylogenetic tree is unstable, with about 95% sequence identity (identities = 1977/2080, 95%; gaps = 47/2080, 2%) to *H*. *modestus* (KC709668) (Fig 1).

### Trypanosomatid prevalence

In 2014, a total of 10,575 individual mosquitoes, consisting of 830 pools, were collected. Of these, 110 pools (13.3%) of six identified mosquito species were positive for trypanosomatids (S2 Table). The most commonly collected mosquito in 2014 was *Ae*. *vexans* with 4420 individuals (41.2%), this mosquito also yielded the highest number of positive pools (n = 33) and the second highest prevalence in the identified mosquito species (21.7%; S2 Table). The highest prevalence of trypanosomatids (23.8%) was found in *Oc*. *sticticus*.

In 2014, individual genetic determination of 2,114 *Cx*. *pipiens* s.l. */ torrentium* mosquitoes in 325 pools revealed that 91.7% (1939 individuals in 221 pools) belonged to the subspecies *Cx*. *pipiens* f. *pipiens*, 2.0% (n = 43; 26 pools) to *Cx*. *pipiens* f. *molestus*, 3.6% (n = 76; 45 pools) to hybrids of the former, and 2.7% (n = 56; 33 pools) to *Cx*. *torrentium*. Altogether trypanosomatid DNA was detected in 25 pools (7.7%) (S1 and S2 Tables). The majority of positives were found in pools of *Cx*. *pipiens* f. *pipiens* (21 pools positive); only one pool of *Cx*. *pipiens* f. *pipiens/molestus* hybrids and three of *Cx*. *torrentium* were positive (Table 1).

**Table 1:** Overall trypanosomatid prevalence (calculated as a minimum infection rate, MIR), and parasite diversity found in mosquitoes of the *Cx*. *pipiens* s.l. and *Cx*. *torrentium*, sampled in Vienna and Eastern Austria in 2014.

mosquito species	n individuals	n pools	n positive pools	% positive pools	MIR	n *T*. *avium* s.l.	n *T*. *culicavium*	n *Crithidia brevicula* and *fasciculata*	n mix of species
***Cx*. *pipiens* f. *pipiens***	1 939	221	21	9.5	1.1	1	9	3	8 (§)
***Cx*. *pipiens* f. *molestus***	43	26	0	0	0				
***Cx*. *pipiens* f. *pipiens/molestus* hybrid**	76	45	1	2.2	1.3				1 (+)
***Cx*. *torrentium***	56	33	3	9.1	5.4		2		1 (X)
**total**	**2 114**	**325**	**25**	**7.7**	**1.2**	**1**	**11**	**3**	**10**

Mixes consisted of (§) *T*. *culicavium* dominant, with unidentified smaller peaks on electropherogram (n = 6); *C*. *brevicula/fasciculata* dominant with unidentified smaller peak on electropherogram (n = 1); unidentified mix (n = 1) (+) mix *Crithidia* sp. possibly *C*. *pragensis* (n = 1) (x) unidentified mix, unable to BLAST (n = 1).

In 2015, nearly twice as many mosquitoes (n = 19,400) were collected, compared to 2014 (n = 10,575). These were divided into 850 pools with a total of 188 pools (22.1%) positive for trypanosomatid DNA (S2 Table). Mosquito species composition did significantly [42] differ between the years, with a much higher proportion of *Cq*. *richiardii* (16%), *Cx*. *pipiens* s.l. / *torrentium* (29.5%) and *Cx*. *martinii* (6.6%) compared to 2014, whereas the proportion of *Ae*. *vexans* and *Oc*. *sticticus* was much lower compare to 2014 (S2 Table). Out of the 188 DNA positive pools, 163 (86.7%) belonged to ten identified mosquito species and 25 (13.3%) pools were of unidentified mosquitoes of the genera *Aedes*/*Ochlerotatus*, *Anopheles*, and *Culex*. The majority of positives (n = 71; 24.4%) were found in *Cx*. *pipiens* s.l. / *torrentium* pools with MIR reaching almost 1%. The highest proportion of trypanosomatid DNA was found in pools of *Aedes vexans* (28.4%; MIR = 1.1%) and *Oc*. *sticticus* (32.3%; MIR = 2.1%). Both *Aedes* species carried predominantly *T*. *theileri/cervi* (S1 Table).

### Trypanosomatid prevalence expressed by minimum infection rate (MIR)

**Fig 2:**
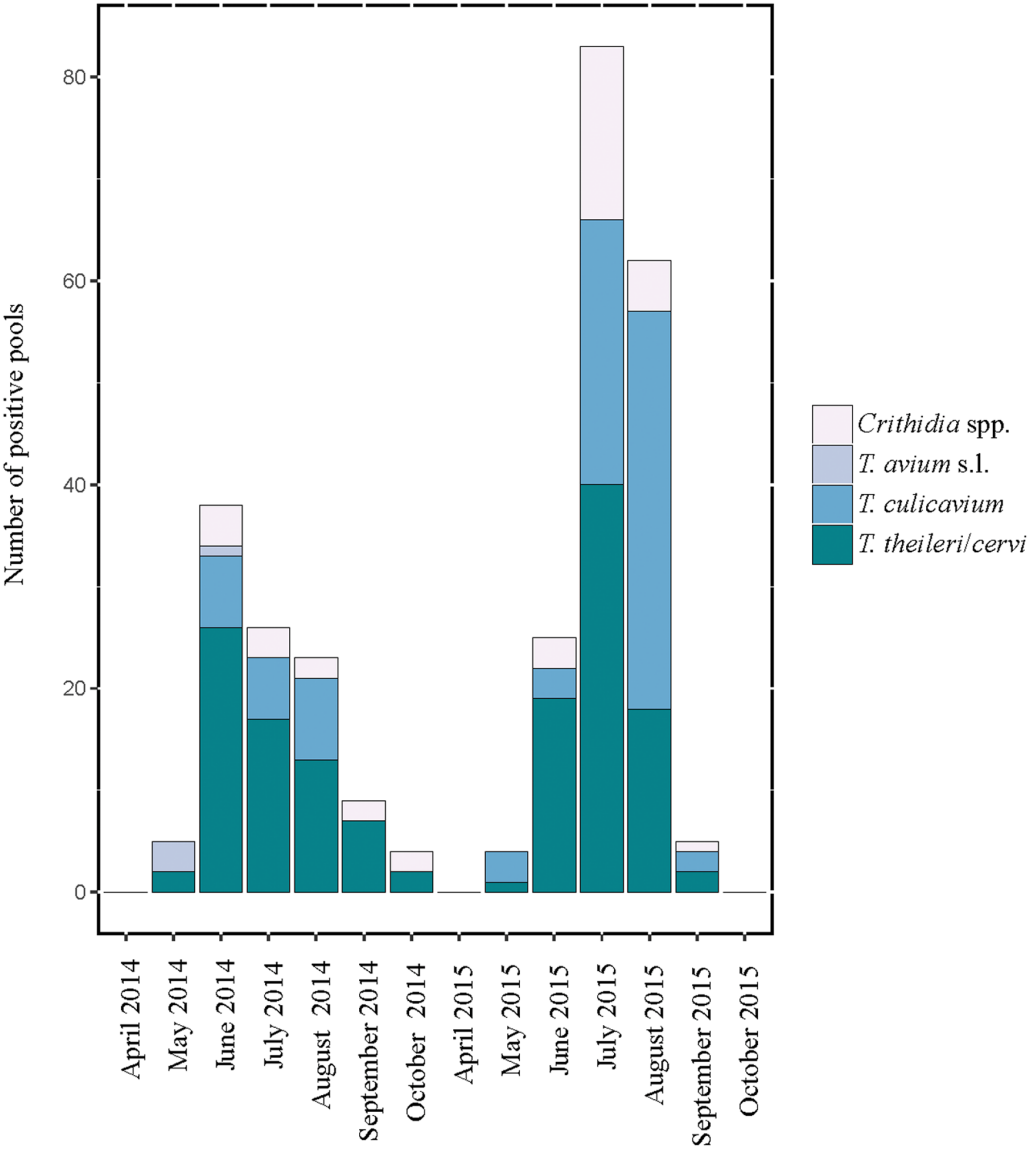
Number of mosquito pools positive for trypanosomatid DNA (trypanosome species, *T*. *avium*, *T*. *culicavium*, and *T*. *theileri*, are shown separately while *Crithidia* spp. infections are combined) according to the sampled months in 2014 and 2015 (Vienna and Eastern Austria).

**Fig 3:**
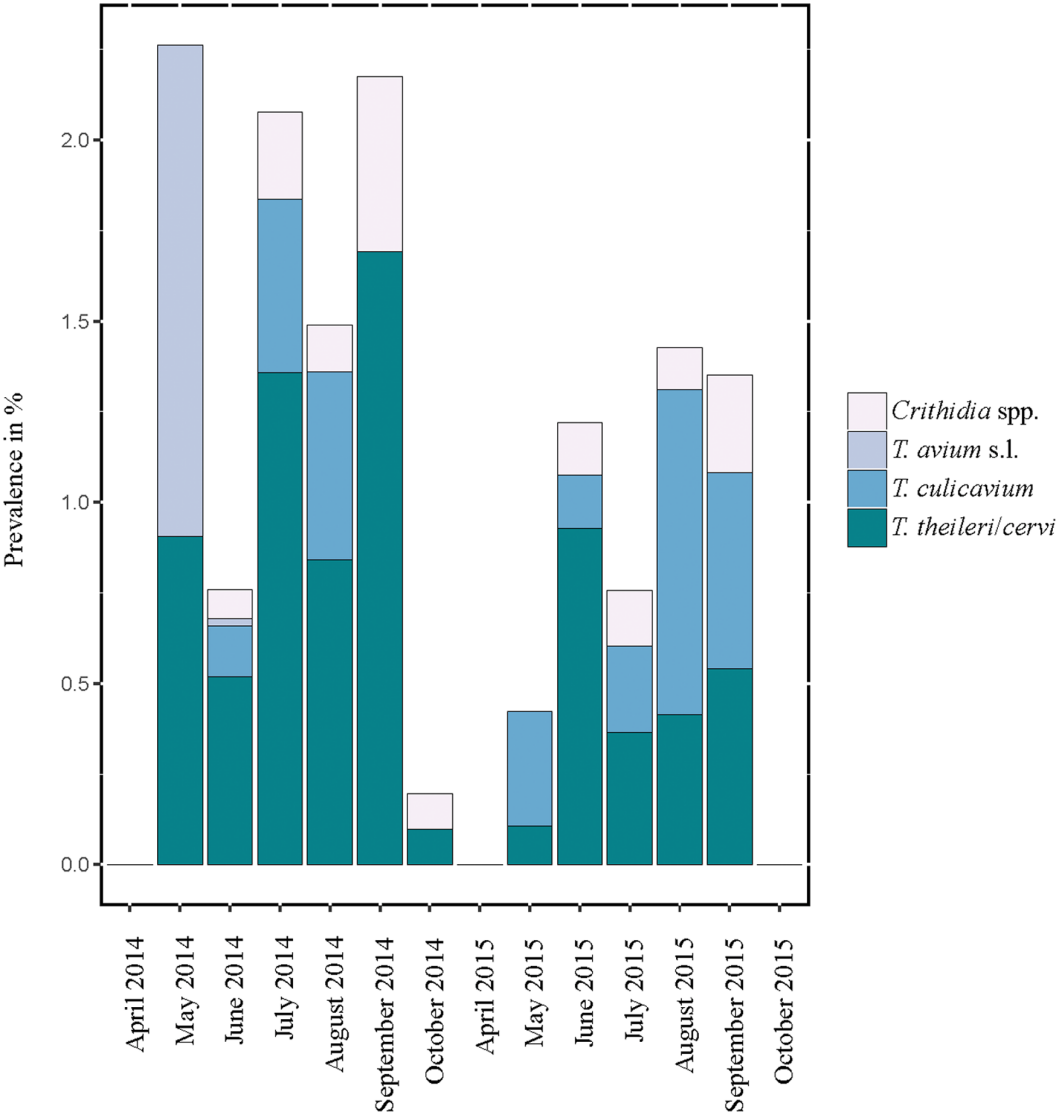
Prevalence of trypanosomatids calculated as minimum infection rate (MIR) (trypanosome species are shown separately while *Crithidia* spp. infections are combined) in mosquitoes according to the sampled months in 2014 and 2015 (Vienna and Eastern Austria).

The minimum infection rate varied between the mosquito species and between the years (S2 Table and Table 1, Figs 2 and 3). The average total MIR (both years and all sampling events) was 0.99%, with the highest prevalence for *Oc*. *geniculatus* (3.9%), *An*. *maculipennis* (1.8%), and *An*. *plumbeus* (1.7%) (S1 Table). The overall highest MIR was found in *Cx*. *martinii* (3.0%) in 2014 and *Oc*. *geniculatus* (16.7%) in 2015 (S2 Table); however the calculated prevalence could be overestimated due to generally low number of tested pools. In 2014, when morphologically undistinguishable mosquitoes of the *Cx*. *pipiens* complex and *Cx*. *torrentium* were identified genetically, it was possible to determine the MIR in the different biotypes comprising this complex (Table 1). Here, *Cx*. *torrentium* presented with the highest MIR (5.4%), followed by *Cx*. *pipiens* f. *pipiens*/*molestus* hybrids (1.3%) and *Cx*. *pipiens* f. *pipiens* (1.1%). No parasite DNA was found in *Cx*. *pipiens* f. *molestus*.

### Monthly changes and differences between 2014 and 2015

The total number and proportion of positive pools in 2014 and 2015 (as well as MIR) was highest in early and mid-summer in both years and became gradually less towards autumn (Fig 2). This is also evident for *T*. *theileri/cervi*, where the numbers (2014) and pool positivity (2015) was highest in June and was gradually tapering off towards October. The trypanosome species *T*. *avium* was only found in 2014, and that year, only in late spring and early summer (May/June). Compared to mammalian trypanosome *T*. *theileri/cervi*, avian *T*. *culicavium* showed the opposite trend; total numbers, pool positivity and MIR increase towards late summer and were highest in August in both years (Figs 2 and 3).

## Discussion

This is the first large scale study of trypanosomatid flagellates in mosquitoes with emphasis on Austria. Our results are of special interest, because the used genetic identification of the *Cx*. *pipiens* complex and *Cx*. *torrentium* mosquito species enabled the determination of trypanosomatid parasites in the morphologically undistinguishable species, biotypes and their hybrids of this complex for the first time. Since we used pools of whole body insects and did not perform microdissections, microscopy or experimental infections, we cannot assert any vector competence and/or host specificity in any of the examined mosquitoes for the detected trypanosome species. However, our results are non-the-less valuable in providing an overview of the dixenous as well as monoxenous trypanosomatid species present in Central Europe.

### Trypanosomatid diversity and prevalence

The trypanosomatid parasites we found in mosquitoes belonged to three trypanosome species (*T*. *theileri* complex, *T*. *culicavium*, and less frequently *T*. *avium* s.l.) and four monoxenous insect species, three of the genus *Crithidia* (*C*. *fasciculata* and *C*. *brevis* were the most frequent, whereas *C*. *pragensis* was found in one pool only) and one of the genus *Herpetomonas* (found in one mosquito pool only). In the previous study performed in neighboring Czechia [16], different bloodsucking dipterans (*Culex* spp., Simuliidae, and Hippoboscidae) were examined for trypanosomatids. *T*. *culicavium*, *T*. *avium*, and *Crithidia brevicula* were detected in both *Cx*. *pipiens* and *Cx*. *modestus*, with overall trypanosomatid prevalence 8.2% and 5.1% in *Cx*. *pipiens* and *Cx*. *modestus*, respectively. In Czechia, the prevalence of *Trypanosoma culicavium* in *Cx*. *pipiens* s.l. and *Cx*. *modestus* varies between 0.3% and 5.4% and between 0.05% and 1.4%, respectively [16, 47]. Similar prevalence of *T*. *culicavium* was detected in *Culex* mosquitoes during our study, ranging from 1 to 3%. Despite the fact that we examined a wider range of mosquito species and a larger amount of individuals than these previous studies in Czechia [16, 47], we only found a small number of *T*. *avium* s.l. positives (only in May and June 2014). *Trypanosoma avium* s.l. is a common parasite in various avian orders worldwide and the prevalence in birds in Europe ranges between 1 to 87.2% [43, 38, 61–63]. Although these parasites have not been studied in Austria in any detail before, a similar range of prevalence would be expected for local birds and the relatively low prevalence we observed in the mosquitoes is surprising. The habitat where the sampling takes place has a great impact on mosquito diversity [64] and the parasites they carry, which is influenced by the available vertebrate host species [16], it is therefore possible that our sampling locations had only low numbers of bird species carrying *T*. *avium* sensu lato.

The parasites *T*. *theileri*, *T*. *cervi*, *T*. cf. *cervi* belong to a complex of species which cannot be resolved using the SSU gene and it is therefore not possible to determine the exact taxonomy of the parasites belonging to this complex found in this study. Further research on the collected material will take more genes into account to resolve this ambiguity.

*Aedes vexans* is a mammalophilic mosquito, and previous studies have shown that wild game animals like red (*Cervus elaphus*) and roe deer (*Capreolus capreolus*) are commonly bitten [65, 66]. In a study in Switzerland, *Ae*. *vexans* blood meals taken from wild game animals were the second most common after blood meals from cattle, with 18.25% of all examined blood meals from red deer, and 5.1% from roe deer [66]. Börstler et al. [65] reported a very similar result for *Ae*. *vexans* in their study on host preferences of different mosquito species in Germany. The information concerning the occurrence of *Trypanosoma theileri*/*cervi* in wild animals, especially cervids, in Central Europe is very limited. The presence of these parasites in Germany and Poland [67, 68] is supported by two trypanosome sequences available in GenBank and obtained from a red deer (*Cervus elaphus*) and fallow deer (*Dama dama*) sampled in Poland. Our frequent findings of *T*. *theileri/cervi* in Austrian mosquitoes (preferably in the genera *Aedes*/*Ochlerotatus* and *Coquillettidia*) is no evidence for the involvement of these mosquitoes in the transmission cycle of the parasite; on the other hand, it proves the abundance of the trypanosomes in the vertebrate hosts (probably game ungulates) in the studied areas.

Monoxenous trypanosomatids infect a broad range of insects, including those of the order Diptera. Due to their limited impact on human and animal health, monoxenous trypanosomatids have received only little attention. Based on PCR screening, three species of the genus *Crithidia*, common parasites of the insect alimentary canal, were detected in mosquito pools. Whereas *C*. *fasciculata*, a well-known laboratory model, infects many mosquito species [69], *Crithidia brevicula* is known mainly from true heteropteran bugs [66, 70]. In Czechia, however, the parasite was found in *Culex* mosquitoes [16]. While the two previous species have been found in mosquitoes repeatedly, the third *Crithidia* species, *C*. *pragensis*, was found only in one pool of *Cq*. *richiardii*. The parasite species was recently described in neighboring Czechia [71] from a brachyceran fly *Cordilura albipes* (Scatophagidae) and our finding therefore extends the possible host spectrum and area of the distribution.

We did not find any *Paratrypanosoma* parasites, repeatedly reported from mosquitoes in the neighboring Czechia [16, 25], but also in the USA [72]. However, in one pool of *Cq*. *richiardii* we have found an unknown species of *Herpetomonas*. This parasite genus is predominately found in dipterans, mainly in brachyceran flies [65], however several studies demonstrated the occurrence of *Herpetomonas* parasites in blood sucking nematoceran insects, specifically in biting midges [17, 18].

One third of all positive mosquito pools examined in this study carried a mix of either two or more different trypanosomatid species. Detecting mixes in this study is a by-product of examining pools of mosquitoes instead of looking at individuals, although the presence of several species, visible as double peaks on the chromatogram, has been found in other studies examining other haematozoa (avian malaria parasites) even in single mosquitoes [73].

### Seasonal changes

During both years, the total trypanosomatid pool prevalence (%) and MIR was highest in the mid of summer and decreased towards autumn. Differences between the years can be explained by climatic differences, since the period April to September in the year 2014 was on average cooler with more precipitation than the same period in 2015. This had an impact on which mosquito species and the numbers of individuals were caught, which was reported in a previous paper [64]. The differences in seasonality between the two dominant trypanosome species are more remarkable. Compared to mammalian *T*. *theileri/cervi*, the total numbers, prevalence (%) and MIR of avian *T*. *culicavium* appeared to increase towards autumn. These noticeable differences can be explained by the different host and vector preferences of both mentioned trypanosomes. While avian *T*. *culicavium* develops in mosquitoes of the genus *Culex*, mammalian *T*. *theileri/cervi* is found mainly in mosquitoes of the genera *Aedes*/*Ochlerotatus* and *Coquillettidia*. Unlike monoxenous trypanosomatids, dixenous trypanosomes infect mosquitoes when sucking blood, and the different behavior and seasonality of various mosquito species/genera may also result in different seasonality and occurrence of transmitted parasites.

*Trypanosoma avium* was only detected in two months (May and June) in 2014, and total numbers, prevalence (%) and MIR were higher in May. It is known that temperature has an impact on the development of trypanosomes in invertebrate hosts. Experiments performed on *T*. *avium* in *Ae*. *aegypti* mosquitoes showed that higher temperatures were detrimental for parasite development and the optimal temperature was around 20 °C. This might be the reason we only observed these parasites during late spring/early summer in 2014. The temperature requirements might be similar for the development of *T*. *theileri/cervi*, although no studies have been performed on this parasite and the only report of seasonal changes in prevalence of *T*. *theileri* in the Northern hemisphere noted an increase in the infection rate of domestic cattle in the state of New York from May to September [74]. In contrast to our detected vector-borne trypanosomes, the monoxenous *Crithidia* spp. appeared evenly distributed over the year, probably due to the horizontal transmission between mosquito hosts via contamination of sugar food sources by parasites.

### Trypanosomatid parasites in mosquitoes of the *Cx*. *pipiens* s.l. / *Cx*. *torrentium*

The second most common mosquitoes caught in this study were species belonging to the morphologically indistinguishable *Cx*. *pipiens* s.l. and *Cx*. *torrentium*. During the previous study, these taxa, sampled in 2014, were identified genetically [54] and this provided us with an opportunity to determine trypanosomatid diversity and prevalence in these *Culex* mosquitoes. The most common trypanosome species we detected in this species complex was *T*. *culicavium*. On the other hand, *Trypanosoma avium* s.l., *Crithidia brevicula* and *C*. *fasciculata* were found only in *Cx*. *pipiens* f. *pipiens*. During our sampling, the most common mosquito of this species group caught was *Cx*. *pipiens* f. *pipiens* and subsequently, the largest total number of trypanosomatids as well as the largest proportion of positive pools was found in this biotype. However, when comparing the MIR of the different biotypes and the hybrids in the species complex, differences are evident. *Culex torrentium* showed the highest MIR, followed by the *Cx*. *pipiens* f. *pipiens*/*molestus* hybrids, while the MIR for *Cx*. *pipiens* was lowest and no trypanosomatids were detected in *Cx*. *pipiens* f. *molestus*. It is unclear if these differences could be explained by the much lower sample size of *Cx*. *pipiens* f. *molestus*, *Cx*. *torrentium* and hybrids or if these mosquitoes in general bite birds infected with *T*. *culicavium* more frequently and therefore have a higher chance of acquiring these parasites.

## Supporting information

S1 Fig Sampling sites for mosquitoes in Eastern Austria during the years 2013−2015. The close-up provides an overview of the city of Vienna where sampling sites were densest. Sites positive for trypanosomatid parasites are marked by stars, negative sites are marked by triangles. The map was constructed using our data and the software: ArcGIS 10.1 (ESRI, Redlands, CA, USA, https://www.esri.com).
(TIF)

S1 Table Overall trypanosomatid prevalence (calculated as a minimum infection rate, MIR), pool positivity, and parasite diversity found in mosquitoes sampled in Vienna and Eastern Austria (2014 and 2015). (DOCX)

S2 Table Occurrence of trypanosomatid DNA in pools of mosquito species collected in Vienna and Eastern Austria (2014 and 2015). (DOCX)
