# Supplementary figures and images for: Trypanosomatid parasites in Austrian mosquitoes
Source: PLoS One. 2018 Apr 19;13(4):e0196052. doi: 10.1371/journal.pone.0196052 (PMC5908168; doi:10.1371/journal.pone.0196052)

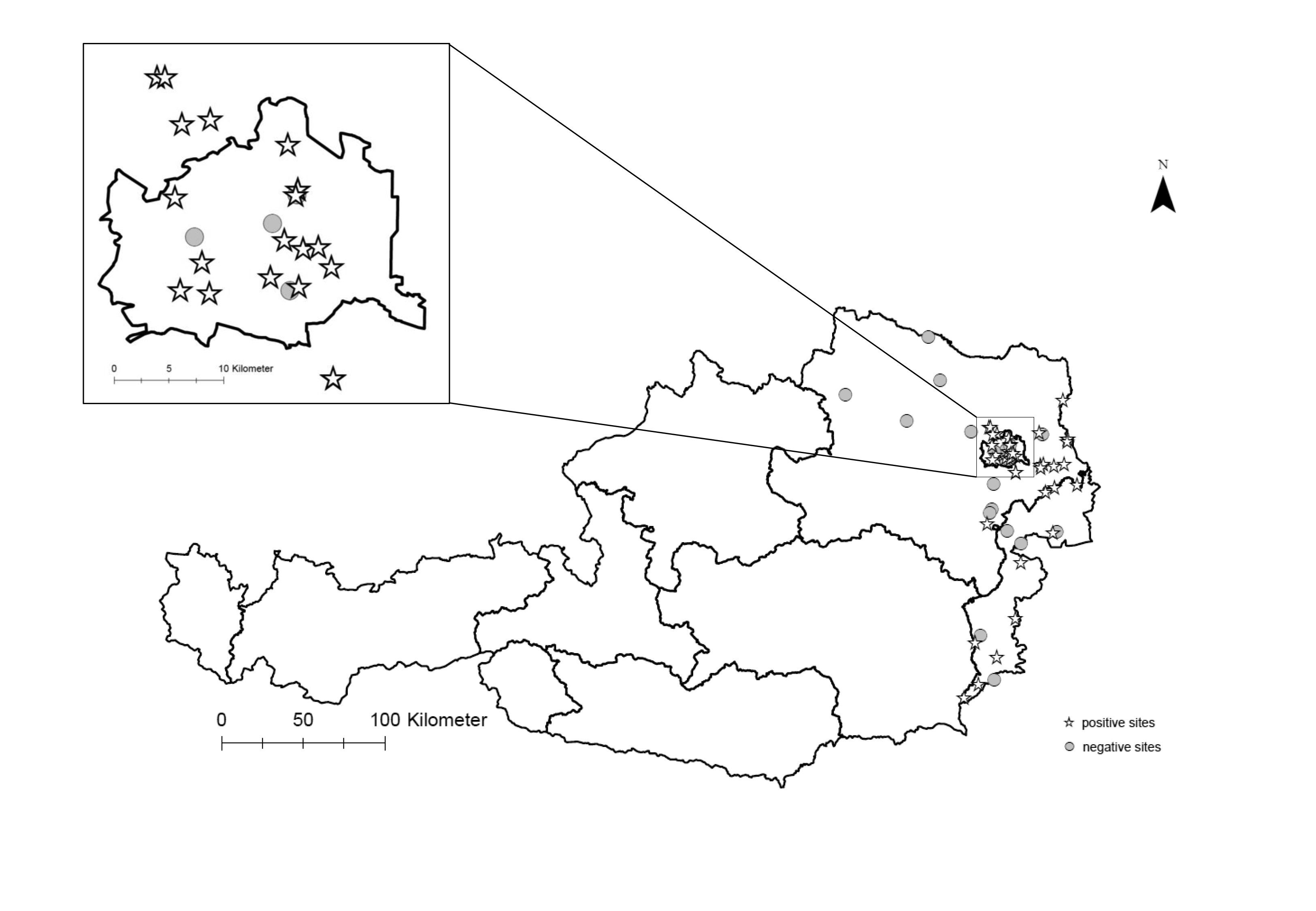

Supplement: S1 Fig — The close-up provides an overview of the city of Vienna where sampling sites were densest. Sites positive for trypanosomatid parasites are marked by stars, negative sites are marked by triangles. The map was constructed using our data and the software: ArcGIS 10.1 (ESRI, Redlands, CA, USA, https://www.esri.com). (TIF) [file pone.0196052.s001.tif]
