# Supplementary material for: Trypanosomatid parasites in Austrian mosquitoes
Source: PLoS One. 2018 Apr 19;13(4):e0196052. doi: 10.1371/journal.pone.0196052 (PMC5908168; doi:10.1371/journal.pone.0196052)
Supplement: S1 Table — (DOCX) [file pone.0196052.s002.docx]

| **mosquito species** | **n individuals** | **n pools** | **n**  **positive pools** | **% positive pools** | **MIR** | **n**  ***T. theileri/cervi*** | **n**  ***T. culiavium*** | **n**  ***T. avium* s.l.** | **n**  ***C.***  ***brevicula*** | **n**  ***C. fasciulata*** | **n**  **C. *pragensis*** | **n**  ***Herpetomonas* sp.** | **n**  **mix of species** |
| --- | --- | --- | --- | --- | --- | --- | --- | --- | --- | --- | --- | --- | --- |
| *Ae. cinereus/geminus* | 381 | 22 | 1 | 4.6 | 0.3 | 1 |  |  |  |  |  |  |  |
| *Ae. vexans* | 5 598 | 214 | 60 | 28.4 | 1.1 | 43 |  | 1 |  | 1 |  |  | 15 ^(1)^ |
| *Aedes/Ochlerotatus* spp. | 1 001 | 87 | 36 | 41.4 | 3.6 | 26 |  | 1 |  |  |  |  | 9 ^(2)^ |
| *An. maculipennis* complex | 55 | 34 | 1 | 2.9 | 1.8 |  |  |  |  |  |  |  | 1 ^(3)^ |
| *An. plumbeus* | 346 | 72 | 7 | 9.7 | 2.0 | 3 |  |  |  |  |  |  | 3 ^(4)^ |
| *Anopheles* spp. | 410 | 36 | 5 | 13.9 | 1.2 | 1 |  |  |  | 1 |  |  | 1 ^(5)^ |
| *Cq. richiardii* | 9 320 | 285 | 51 | 17.9 | 0.6 | 14 |  |  | 6 | 6 | 1 | 1 | 23 ^(6)^ |
| *Cx. martinii* | 1 062 | 44 | 2 | 4.5 | 0.1 |  |  |  | 1 |  |  |  | 1 ^(7)^ |
| *Cx. modestus* | 63 | 8 | 1 | 12.5 | 1.6 |  | 1 |  |  |  |  |  |  |
| *Cx. pipiens* s.l./ *torrentium* | 9 292 | 616 | 96 | 15.6 | 1.0 | 1 | 60 | 1 |  | 2 |  |  | 31 ^(8)^ |
| *Culex.* spp*.* | 406 | 75 | 12 | 16.0 | 3.0 |  | 8 |  | 1 |  |  |  | 3 ^(9)^ |
| *Oc. geniculatus* | 26 | 19 | 1 | 5.3 | 3.9 | 1 |  |  |  |  |  |  |  |
| *Oc. sticticus* | 1 620 | 94 | 25 | 26.6 | 1.5 | 15 |  |  |  |  |  |  | 10 ^(10)^ |
| Other taxa* | 395 | 76 | 0 | 0 | 0 |  |  |  |  |  |  |  |  |
| **Total** | **29 975** | **1 680** | **298** | **17.7** | **1** | **105** | **69** | **3** | **8** | **10** | **1** | **1** | **98** |

* other taxa: *Anopheles algeriensis, An. claviger*, *An. hyrcanus, Cs. annulata*, *Cx. territans*, *Oc. cantans*, *Oc. caspius*, *Oc. cataphylla*, *Oc. communis*, *Oc. flavescens*, *Oc. intrudens*, *Oc. japonicus*, *Oc. leucomelas*, *Oc. rusticus* and *Uranotaenia unguiculata*.

**Mixes consisted of (1) *C. brevicula/fasciculata* dominant with unidentified smaller peaks on electropherogram (n=1); *T. theileri/cervi* dominant with unidentified smaller peaks on electropherogram (n=14), (2) *T. theileri/cervi* dominant with unidentified smaller peaks on electropherogram (n=9), (3) *T. theileri/cervi* dominant with unidentified smaller peaks on electropherogram (n=1), (4) *C. brevicula/fasciculata* dominant with unidentified smaller peaks on electropherogram (n=2); *T. theileri/cervi* dominant with unidentified smaller peaks on electropherogram (n=1) (5) *T. theileri/cervi* dominant with unidentified smaller peaks on electropherogram (n=2) (6) one case possibly *T. culicavium* with unidentified other sequence; *C. brevicula/fasciculata* dominant with unidentified smaller peak on electropherogram (n=10); *T. theileri/cervi* dominant with unidentified smaller peaks on electropherogram (n=12) (7) *C. brevicula/fasciculata* dominant with unidentified smaller peak on electropherogram (n=1) (8) *T. culicavium* dominant, with unidentified smaller peaks on electropherogram (n=22); *C. brevicula/fasciculata* dominant with unidentified smaller peak on electropherogram (n=2); mix *Crithidia* sp. possibly *C. pragensis* (n=2)*;* unidentified mix, unable to BLAST (n=5) (9) *T. culicavium* with other unidentified sequence (n=3) (10) *T. theileri/cervi* dominant with unidentified smaller peaks on electropherogram (n=10).**
