# Supplementary material for: Trypanosomatid parasites in Austrian mosquitoes
Source: PLoS One. 2018 Apr 19;13(4):e0196052. doi: 10.1371/journal.pone.0196052 (PMC5908168; doi:10.1371/journal.pone.0196052)
Supplement: S2 Table — (DOCX) [file pone.0196052.s003.docx]

|  | **2014** | | | | | **2015** | | | | |
| --- | --- | --- | --- | --- | --- | --- | --- | --- | --- | --- |
| **mosquito species** | **n**  **indiv.** | **n**  **pools** | **n**  **pos.**  **pools** | **%**  **pos.**  **pools** | **MIR** | **n**  **indiv.** | **n**  **pools** | **n**  **pos.**  **pools** | **%**  **pos.**  **pools** | **MIR** |
| *Ae. cinereus/*  *geminus* | 348 | 16 | 0 | 0 | 0 | 33 | 6 | 1 | 16.7 | 3.0 |
| *Ae. vexans* | 4 420 | 152 | 33 | 21.7 | 0.7 | 1 178 | 62 | 27 | 43.6 | 2.3 |
| *Aedes/Ochlerotatus* spp*.* | 784 | 60 | 21 | 35.0 | 2.7 | 217 | 27 | 15 | 55.6 | 6.9 |
| *An. maculipennis* complex | 14 | 11 | 0 | 0 | 0 | 41 | 23 | 1 | 4.4 | 2.4 |
| *An. plumbeus* | 150 | 30 | 2 | 6.7 | 1.3 | 196 | 42 | 4 | 9.5 | 2.0 |
| *Anopheles* spp. | 22 | 8 | 2 | 25.0 | 9.1 | 388 | 28 | 3 | 10.7 | 0.8 |
| *Cq. richiardii* | 1 287 | 68 | 5 | 7.4 | 0.4 | 8 033 | 217 | 46 | 21.2 | 0.6 |
| *Cx. pipiens* s.l./ *torrentium* | 2 114 | 325 | 25 | 7.7 | 1.2 | 7 178 | 291 | 71 | 24.4 | 1.0 |
| *Cx. martinii* | 66 | 11 | 2 | 18.2 | 3.0 | 996 | 33 | 1 | 3.0 | 0.1 |
| *Cx. modestus* | 2 | 2 | 0 | 0 | 0 | 61 | 7 | 1 | 14.3 | 1.6 |
| *Culex* spp. | 131 | 41 | 5 | 12.2 | 3.8 | 275 | 34 | 7 | 20.6 | 2.6 |
| *Oc. geniculatus* | 20 | 13 | 0 | 0 | 0 | 6 | 6 | 1 | 16.7 | 16.7 |
| *Oc. sticticus* | 1 139 | 63 | 15 | 23.8 |  | 481 | 31 | 10 | 32.7 | 2.1 |
| other taxa* | 78 | 30 | 0 | 0 | 0 | 839 | 100 | 0 | 0 | 0 |
| **total** | **10 575** | **830** | **110** | **13.3** | **1.0** | **19 400** | **850** | **188** | **22.1** | **0.9** |

* other taxa: *Anopheles algeriensis, An. claviger*, *An. hyrcanus, Cs. annulata*, *Cx. territans*, *Oc. cantans*, *Oc. caspius*, *Oc. cataphylla*, *Oc. communis*, *Oc. flavescens*, *Oc. intrudens*, *Oc. japonicus*, *Oc. leucomelas*, *Oc. rusticus* and *Uranotaenia unguiculata*.
